# Catatonia-Induced Saddle Pulmonary Embolism

**DOI:** 10.7759/cureus.14000

**Published:** 2021-03-19

**Authors:** Brittany S Maner, Jasbir Singh, Homero Camacho, Andrew K Jenson

**Affiliations:** 1 Psychiatry, Kern Medical, Bakersfield, USA; 2 Psychiatry, University of California Los Angeles-Kern Medical Center, Bakersfield, USA; 3 Psychiatry, American University of the Caribbean, Bakersfield, USA

**Keywords:** catatonia, pulmonary embolism, deep venous thrombosis, schizoaffective disorder, lorazepam, retarded-type

## Abstract

In this case report, we highlight a patient with catatonia secondary to schizoaffective disorder, depressive type. This patient developed a bilateral deep venous thrombosis progressing to a saddle pulmonary embolism without any predisposing factors to hypercoagulability other than immobility and obesity. The goal of this case report is to increase monitoring and prophylactic treatment for deep venous thrombosis in patients with catatonia, retarded type.

## Introduction

Pulmonary embolism (PE) is a potentially life-threatening complication of deep venous thromboses (DVTs). Thrombosis of blood can be caused by a state of hypercoagulability, vessel wall injury, and stasis of blood commonly referred to as Virchow’s triad. There are many factors that can lead to hypercoagulability including genetic factors, acquired factors, environmental factors, mixed factors, and others not well established. One of the most important acquired factors that require focus for patients with mental illness is immobility [1]. Pulmonary emboli are commonly caused by dislodged DVTs provoked by the contractile movement of the lower extremity muscles causing veins to pump blood and emboli back to the heart and pulmonary vasculature. Pulmonary emboli have a high rate of mortality, especially in patients with illnesses causing retarded-type catatonia [2].

Catatonia is a behavioral syndrome with the incapacity to move properly, which occurs in various psychiatric and non-psychiatric illnesses. In fact, 10% of acutely ill psychiatric patients in an inpatient psychiatric unit were found to have catatonia, most of who presented with mood disorders [3]. It is described in the Diagnostic and Statistical Manual of Mental Disorders 5 (DSM-5) as having “three or more of 12 psychomotor features in the diagnostic criteria for catatonia associated with another mental disorder and catatonic disorder due to another medical condition.” These symptoms include stupor, catalepsy, waxy flexibility, mutism, negativism, posturing, mannerism, stereotypy, agitation not influenced by external stimuli, grimacing, echolalia, and echopraxia. The severity of catatonia can be measured through the 23-item Bush-Francis Catatonia Rating Scale, which measures the previously mentioned signs and symptoms of catatonia. Each item is scored 0-3 depending on severity. The higher the score, the more unstable the patient will be [4]. There are three subtypes: retarded, excited, and malignant. The retarded type is described as having an inability to move, staring, posturing, negativism, and mutism [5]. For centuries, schizophrenia has been divided into two different groups: catatonia group and non-catatonia group [6]. Catatonia is now described in the DSM-5 as a separate condition most commonly associated with mood disorders such as bipolar I and II; however, it is also associated with schizophrenia spectrum disorders and autism spectrum disorder [3]. There are many complications to catatonia including malnutrition, dehydration, contractures, pressure ulcers, DVTs, and PEs [7]. Of the three subtypes of catatonia, the retarded subtype is the most susceptible to DVTs and pulmonary emboli due to immobility, and hemoconcentration secondary to decreased food and water intake [2]. Catatonia, retarded type is a dangerous and life-threatening presentation of schizoaffective disorder. Due to their stuporous state, these patients have the potential to develop DVTs for a prolonged period of time. The lorazepam challenge is one of the major forms of diagnosing patients with catatonia. A patient is given 1-2 mg of lorazepam intramuscularly, intravenously, or orally and observed for rapid resolution of symptoms after a 20 to 30-minute time period [5]. Yet, without proper anticoagulation, patients with catatonia and DVTs on the lorazepam challenge can quickly develop pulmonary emboli which can lead to their demise. A 2016 study proposes that patients with catatonia have a 25% incidence of developing DVTs [2]. However, there are only two documented cases of patients with retarded-type catatonia developing DVTs and subsequent PEs. These patients may be more susceptible to a delayed diagnosis and thus decreased standard of care, leading to worse health outcomes. Catatonia and its complications, namely DVT and PE, are important clinical scenario to consider in at-risk populations. Our case can bring proper awareness of the risk of DVT and PE in a patient presenting with retarded subtype of catatonia. The Kern Medical Institutional Review Board approved the study protocol 19067 where we will elaborate on the effect of a retarded-type catatonic patient suffering from a massive PE. Consent to publish the case history was obtained from the patient.

## Case presentation

A 19-year-old African American female with a previous diagnosis of catatonia secondary to schizoaffective disorder, post-traumatic stress disorder, hypothyroidism, BMI of 44.4, and history of deep venous thrombosis. She previously had over five episodes of retarded-type catatonia with hospitalizations for being gravely disabled due to decreased food and water intake for various but prolonged periods of time. In the state of California, “gravely disabled” is defined as being unable to utilize food, clothing, and shelter, which is grounds for admission into inpatient psychiatric unit. Her first episode of catatonia began prior to April 2018 according to hospital and outpatient records. She was first diagnosed with a left common femoral venous thrombosis by Hematology and Oncology in April 2018. The patient was then investigated for having risk factors for inherited hypercoagulability where her prothrombin gene mutation, protein C activity, protein S activity, antithrombin III activity, factor V Leiden mutation, and lupus anticoagulant, all returned within normal range; no mutations present. Previous inpatient and outpatient records did not report the use of oral contraceptive pills or tobacco use history, symptoms of irritable bowel disease, and other acquired forms of hypercoagulability. In April 2018, she was prescribed rivaroxaban by Hematology and Oncology for DVT treatment and decreasing risk for further DVT formation. However, she was lost to follow up and was reported medication non-adherent.

In August 2019, the patient was admitted to the inpatient psychiatric unit. Her catatonia was displayed with stupor, mutism, staring, and immobility. She was referred from a group home five days after being medically cleared from another hospital as gravely disabled for a duration of five days. The patient presented in a mute state refusing psychiatric evaluation, interview, or following any instructions. A preliminary diagnosis of catatonia was made with a Bush-Francis Rating Scale of 14/69. A lorazepam challenge was performed. Thirty minutes after administration of 1 mg lorazepam by mouth, she was significantly more verbal, mobile, and appropriately responding to jokes made by her peers. Her subsequent Bush-Francis Rating Scale decreased to 5/69. She stated that she did not recall the events leading up to her hospitalization.

**Figure 1:**
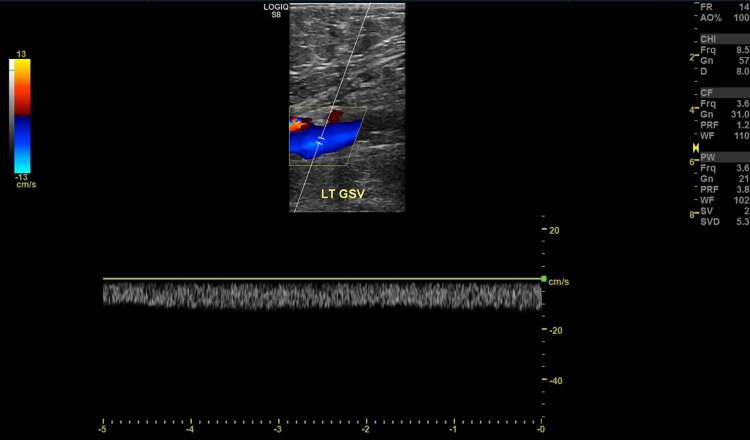
Doppler waveforms and partial occlusion within the left great saphenous vein.

**Figure 2:**
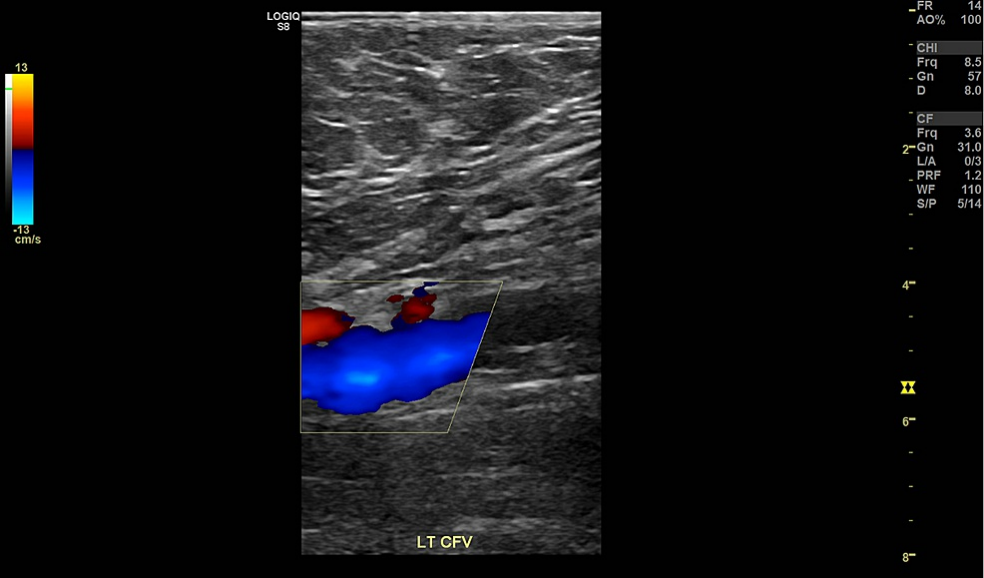
Partial occlusion of the left common femoral vein.

**Figure 3:**
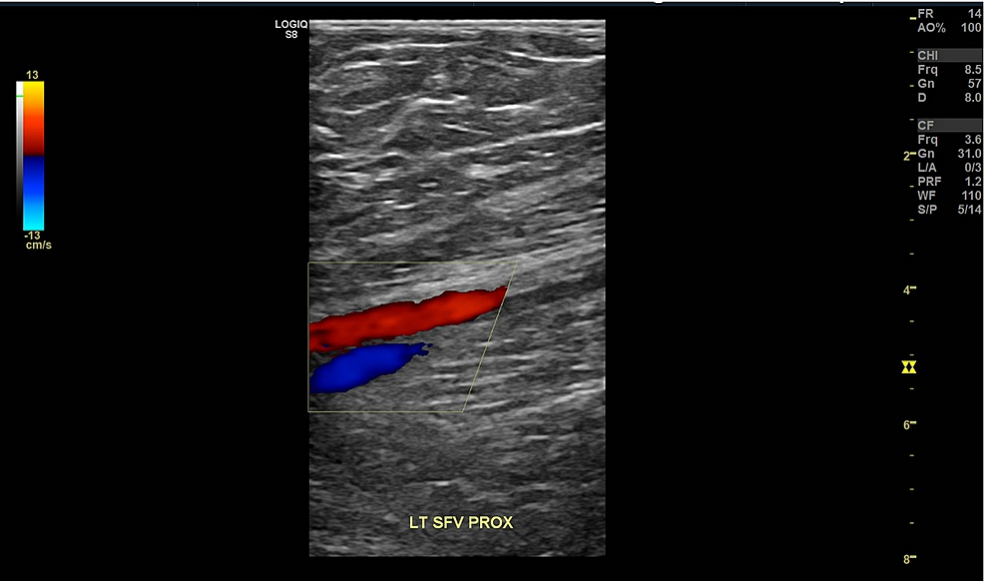
Partial occlusion of the left proximal superficial femoral vein.

**Figure 4:**
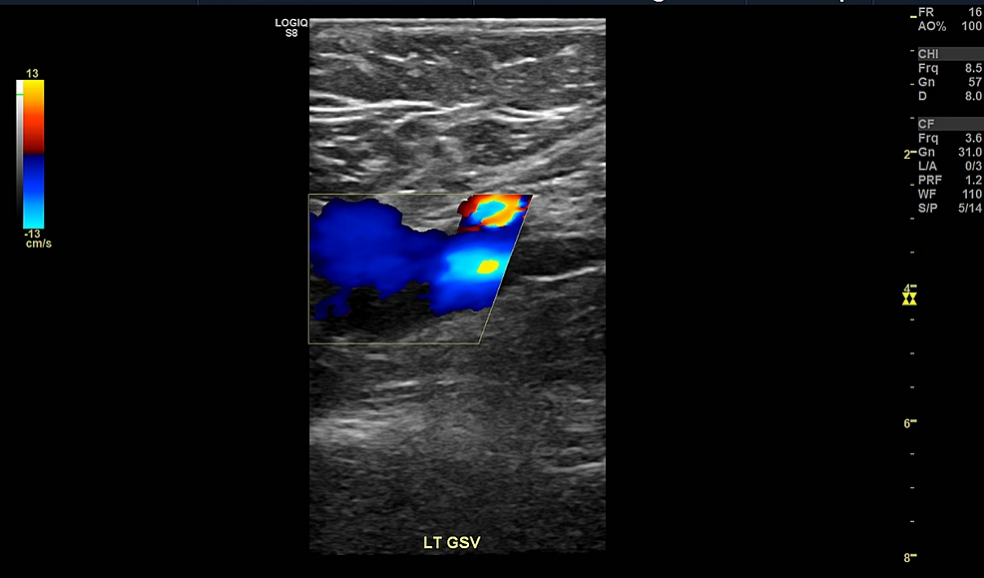
Partial occlusion within the left great saphenous vein.

During this admission, risperidone and valproic acid were initiated for treatment of schizoaffective disorder, levothyroxine was initiated for hypothyroidism, and bilateral lower extremity Doppler ultrasound was ordered to assess previously diagnosed left common femoral venous thrombosis. The patient was found to have non-occlusive DVTs extending from the femoral and popliteal veins and their branches (Figures 1-4). The internal medicine team was immediately consulted and recommended rivaroxaban for anticoagulation, prevention of further progression of her bilateral venous thromboses, and PE prophylaxis.

**Figure 5:**
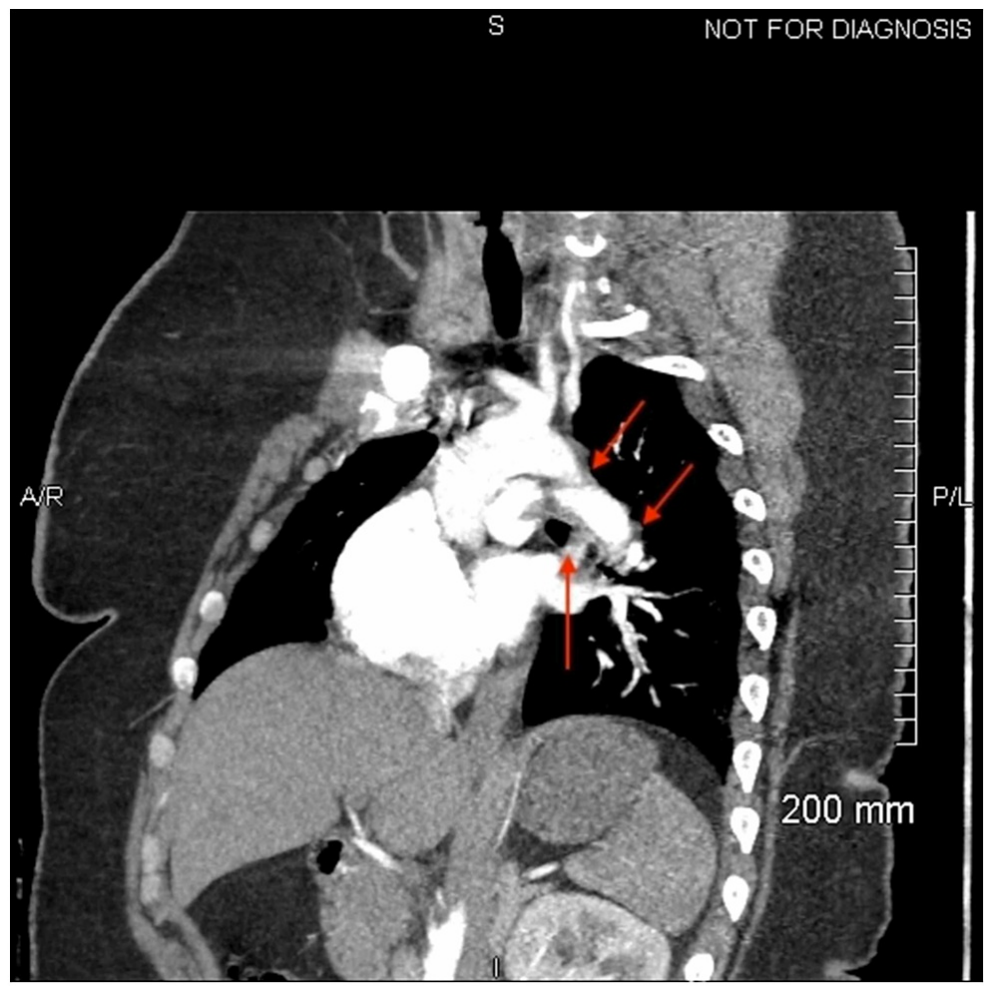
Sagittal view of the CT angiogram displaying a pulmonary embolism. The red arrows indicate the location of the embolis.

**Figure 6:**
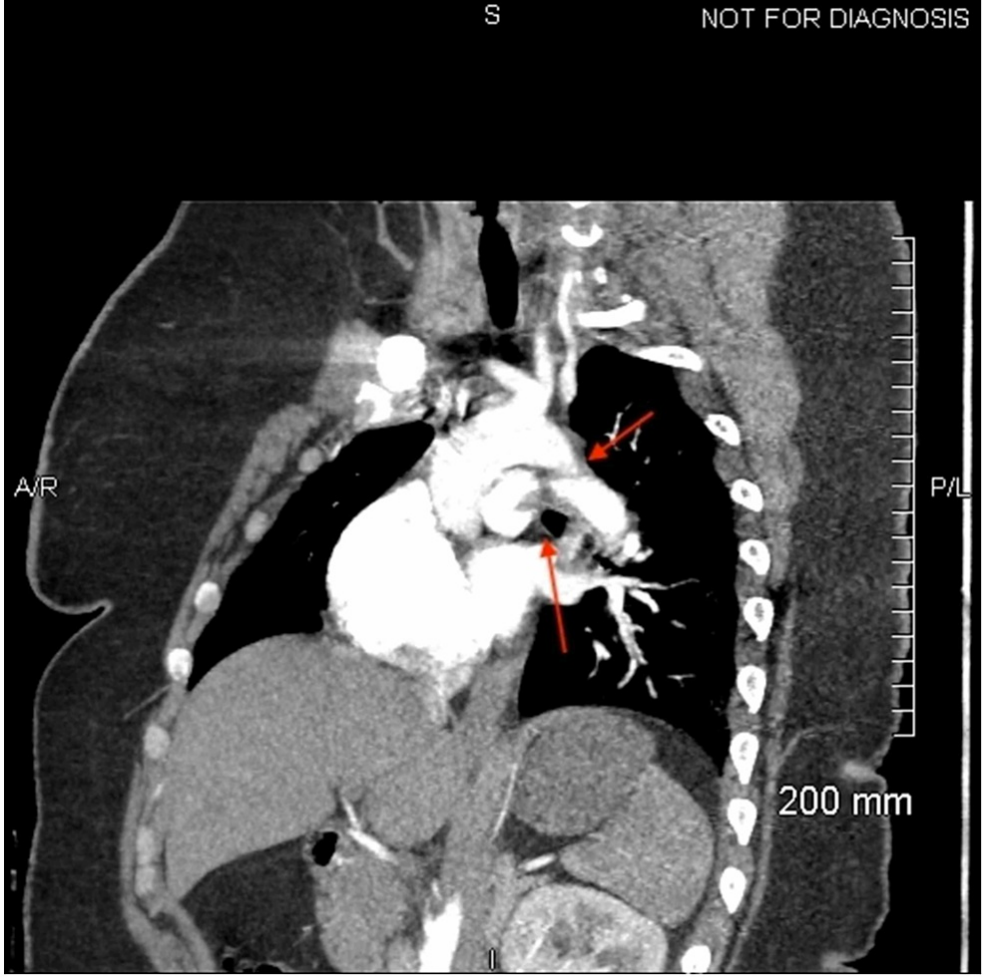
Inferior and sagittal view of a pulmonary embolism within the proximal right and left main pulmonary arteries indicated by the red arrows.

Within six hours, the patient began complaining of dyspnea. Vital signs were taken and showed saturated oxygen levels of 93%, blood pressure of 83/61 mmHg, heart rate of 124 beats per minute, respiratory rate of 40 breaths per minute, and repeat vital signs showed similar results. The internal medicine team initiated the induction of heparin and performed a computed tomography angiography (CTA). The CTA revealed a saddle embolism within the main pulmonary artery. Additionally, there were filling defects within the proximal right and left main pulmonary arteries extending into the lobar and segmental branches; it also displayed enlargement of the right ventricle. These findings suggested the right heart strain (Figures 5, 6). The patient was then transferred to the intensive care unit (ICU). Early the next morning, mechanical thrombectomy was performed to remove the embolism as well as interventional radiology guided inferior vena cava filter placement. While in the ICU, the patient’s psychiatric state deteriorated back to catatonic symptoms. The patient was then stabilized and transferred to the medicine floor for further observation, heparin level, prothrombin time, and partial thromboplastin time monitoring were done prior to transferring back to the inpatient psychiatric unit. She was transferred to the inpatient psychiatric unit five days later, in a stuporous, mute, and immobile catatonic state. In the unit, she was adherent to psychotropic and medical medications and was given 1 dose of lorazepam 1 mg with an observed resolution of catatonic symptoms.

## Discussion

Retarded-type catatonia causes stupor, a state of immobility that leads to blood stasis and the ability for clots to form in patients that are affected. Untreated pulmonary emboli are associated with mortality as high as 30% where up to 10% of patients with PEs die suddenly [8]. Many complications, including right heart strain as found in our patient, can be a cause of death or decreased quality of life. However, medicine often neglects to acknowledge the impact of catatonia secondary to psychiatric disease as a major risk factor for blood stasis and hypercoagulability.

Few case reports have discussed the importance of using anticoagulant prophylaxis in patients with catatonia, retarded type especially prior to administration of lorazepam. A case by King et al. involved a 44-year-old female patient with schizoaffective disorder who suffered from bilateral pulmonary emboli and right heart strain due to sedation and previous psychiatric hospitalization. In fact, this case report recommends screening and assessments for sedentary psychiatric patients [9]. Furthermore, the study by Warriach et al., demonstrates that patients with catatonia secondary to schizophrenia can continue electroconvulsive therapy while on anticoagulants depending on the size and the location of their thromboemboli without complications of bleeding [10]. This supports that prior to the administration of lorazepam, catatonic patients may have similar outcomes and be able to tolerate anticoagulant therapy.

In our case report, the patient showed significant improvement in her catatonic symptoms after administration of lorazepam, but quickly after, developed a large PE. Our limitations included the bilateral lower extremity studies that were performed after administration of lorazepam, which delayed anticoagulant treatment, increasing the risk for DVT migration to the lungs. We were unable to obtain the images of the previously diagnosed DVT Doppler reading and the most recent right Doppler images. We were also limited by the reported lack of medication adherence with previously diagnosed DVT. However, this shows the importance of prophylactic treatment with anticoagulant therapy and early investigative studies with retarded-type catatonic patients especially those who have had many catatonic episodes and/or have been in a stuporous state for a prolonged period of time. Lorazepam is an effective treatment for patients with catatonia, retarded type. Nevertheless, these patients’ increased risk for DVT needs to be assessed for a proper treatment plan outside of medications for the catatonia causing diseases. Patients with catatonia would benefit from anticoagulant medications to prevent complications as well as decrease morbidity and mortality. A 2013 study indicates a significantly increased risk of DVT in patients with retarded subtype catatonia. The catatonic patients were compared to restrained non-catatonic patients. The incidence of DVT was 25.3% in catatonic patients. Moreover, DVT in retarded-type catatonic patients compared to restrained non-catatonic patients was significantly higher with an incidence rate of 35.7% in retarded type catatonic patients in comparison to 10.6% in restrained non-catatonic patients [2]. This information shows the increased risk of DVTs and possible PEs in patients with catatonia, retarded subtype. The literature recommends increasing exercise and giving high dose heparin for psychiatric patients, such as patients with catatonia retarded type that are at risk for DVTs and PEs [11].

## Conclusions

This case report can add to the body of knowledge surrounding DVTs and their subsequent complications in patients with catatonia, retarded type. Our patient in particular presented with high clinical suspicion of catatonia secondary to schizoaffective disorder, depressive type, with a past medical history of previous episodes of DVT. She was adequately treated for her PE using anticoagulation, embolectomy, and an IVC filter. However, if patients with multiple episodes of catatonia are treated with anticoagulation therapy for PE prophylaxis, the mortality rate in this patient population may potentially decrease.
